# Statewide Assessment of Public Park Accessibility and Usability and Playground Safety

**DOI:** 10.3390/ijerph23010139

**Published:** 2026-01-22

**Authors:** Iva Obrusnikova, Cora J. Firkin, Riley Pennington, India Dixon, Cole Bilbrough

**Affiliations:** 1 Department of Health Behavior and Nutrition Sciences, University of Delaware, Newark, DE 19713, USA; cfirkin@lincoln.edu; 2 Department of Kinesiology and Applied Physiology, University of Delaware, Newark, DE 19713, USA; rnp@udel.edu; 3 Department of Medical and Molecular Sciences, University of Delaware, Newark, DE 19713, USA; imd45@drexel.edu; 4 English Department, University of Delaware, Newark, DE 19716, USA; colebil@udel.edu

**Keywords:** accessibility, disability inclusion, universal design, public parks, playgrounds, built environment, usability, safety, health equity

## Abstract

**Highlights:**

**Public health relevance—How does this work relate to a public health issue?**

- Statewide evaluation of accessibility, usability, and safety in public parks with playgrounds as community settings for physical activity;
- Focus on disability-inclusive access to public environments that influence physical activity, social participation, and equitable health opportunities.

**Public health significance—Why is this work of significance to public health?**

- Identifies structural and environmental gaps, such as transit, crossings, curb ramps, restrooms, trails, and play features, that restrict safe, independent use of parks and playgrounds for people with disabilities;
- Demonstrates how park and playground accessibility and safety conditions may shape disparities in physical activity and community engagement.

**Public health implications—What are the key implications or messages for practitioners, policy makers and/or researchers in public health?**

- Identifies actionable priorities for improving transit access, pedestrian infrastructure, amenities, inclusive play design, and communication support;
- Provides evidence to guide equitable reinvestment and integration of disability inclusion in planning, transportation, and parks and recreation policy and practice.

**Abstract:**

Accessible and inclusive community environments support physical activity and health equity for people with disabilities, yet gaps in design, maintenance, and communication limit safe, independent use. This statewide cross-sectional audit assessed park accessibility and usability and playground safety in publicly accessible, non-fee-based Delaware community parks with playgrounds. Fifty stratified sites were evaluated using the Community Health Inclusion Index and the America’s Playgrounds Safety Report Card by trained raters with strong interrater reliability. Descriptive analyses summarized accessibility, usability, communication, and safety features by county, with exploratory urban-suburban/micropolitan contrasts. Most sites provided wide, smooth paths, shade, and strong playground visibility, but foundational accessibility varied. Only 30% had a nearby transit stop, fewer than 10% of crossings included auditory or visual signals. Curb-ramp completeness was inconsistent, with detectable warnings frequently absent. Restrooms commonly lacked low-force doors or operable hardware, and multi-use trails often had obstacles or lacked wayfinding supports. Playground accessibility features were present at approximately two-thirds of sites, and 62% were classified as safe, although 10% were potentially hazardous or at-risk. Higher playground accessibility scores were strongly associated with lower life-threatening injury risk. Overall, gaps in transit access, pedestrian infrastructure, amenities, and communication support limit equitable, health-supportive park environments and highlight priority improvement areas.

## 1. Introduction

About 26% of U.S. adults (>70 million) report living with a disability [1]. In Delaware, the prevalence is similar at 25.1% (205,381 adults) [2]. Individuals with disabilities experience disproportionately higher rates of preventable chronic conditions; recent data indicate that 80% of adults with disabilities live with at least one chronic illness, including cardiovascular disease, diabetes, obesity, and arthritis [3,4]. These disparities arise not only from impairment-related factors but also from ongoing systemic barriers in community environments. These barriers include limited physical access, fewer opportunities for social participation, and insufficient support for health promotion, which limit opportunities for physical activity among people with disabilities [3,5]. National surveillance data show that adults with disabilities are less physically active than their peers without disabilities. This difference contributes to higher body mass index, increased risk of inactivity-related conditions, and greater health care and societal costs [6,7].

Participation in physical activity depends on the availability of supportive community environments [7]. Parks, playgrounds, and trails are key settings for leisure-time physical activity and social interaction. Design features that support accessibility and inclusion determine whether these settings can be used equitably and foster a sense of belonging for people of all abilities [5,8]. When designed inclusively, these places enable individual use and foster social connection and community engagement. In this context, accessibility refers to removing physical and structural barriers so that places can be used safely and independently by people with disabilities [9]. Inclusion extends beyond the physical environment to coordinated supports across the built environment, equipment, programs, staff, and policies that enable equitable participation in shared community settings [10]. These concepts are rooted in theoretical and policy frameworks that guide how environments are evaluated and how improvements are planned.

This study is grounded in two complementary frameworks. The World Health Organization’s International Classification of Functioning, Disability, and Health (ICF) [11] conceptualizes disability as arising from interactions between a person’s health condition and contextual factors. Within this framework, environmental factors, such as the accessibility and social openness of community settings, can act as barriers or facilitators that influence functioning, participation, and health. When environments reduce barriers and support participation, they can operate as inclusive places, even though the ICF does not explicitly define inclusion as a construct. Complementing this global framework, U.S. legislation offers concrete, enforceable standards for accessibility implementation. The Americans with Disabilities Act (ADA) [12] provides the legal basis for accessibility, while the ADA Accessibility Guidelines (ADAAG) specify technical requirements for routes, entrances, parking, restrooms, and signage [9]. The 2023 Public Rights-of-Way Accessibility Guidelines (PROWAG) expand these standards to sidewalks, crosswalks, curb ramps, transit stops, and on-street parking [13], features that are critical for park access across Delaware’s community settings.

Although U.S. accessibility standards offer a strong legal and technical foundation, access to community recreation settings remains constrained by noncompliance, aging infrastructure, limited maintenance, and slow adoption of universal design, all of which contribute to inequitable use [5]. Universal design emphasizes flexible, adaptable environments that anticipate diverse user needs and extends beyond minimum compliance [14]. However, most existing park audits have primarily focused on structural accessibility and selected amenities, with comparatively limited attention to usability, communication, and safety from a disability inclusion perspective [8,10,15]. National and international audits consistently report persistent accessibility gaps, particularly in routes, surfacing, inclusive play components, and wayfinding, indicating that compliance-focused design has not consistently translated into usable environments [8,10,16,17]. Even where accessible features are present, deferred maintenance and limited application of universal design can undermine long-term functional usability [16,17]. Safety and maintenance indicators remain underrepresented in most accessibility audits.

Recent scholarship has further emphasized that accessibility compliance alone is insufficient to ensure disability-inclusive public open spaces, calling for integrated consideration of usability, safety, communication, and other environmental determinants of functional use and inclusion. A 2025 systematic review highlights persistent gaps between formal accessibility standards and functional inclusion in outdoor recreational environments and underscores the need for empirical, context-specific assessments that examine how accessibility and safety intersect in practice [18]. Complementary participatory research illustrates that usability and play value often diverge from design intentions grounded solely in regulatory compliance, reinforcing the importance of evaluating environmental features that support functional use, even when user perspectives are not directly assessed [19].

Building on these limitations, several empirical gaps remain. First, many prior studies have examined accessibility at isolated points (e.g., entrances, routes, or play areas) or have focused primarily on structural compliance, without assessing how external access, on-site amenities, and communication features function together to support independent park use [10,15,20]. Second, usability and communication features (e.g., benches, rest areas, wayfinding supports, and accessible information) have been infrequently assessed, despite their influence on comfort, navigation, and perceived inclusion for people with disabilities [5,10]. Third, playground safety has typically been evaluated independently of accessibility, resulting in limited empirical evidence on whether parks that perform well on accessibility indicators also provide safer play environments for children with disabilities [15,20,21]. Methodologically, much of the existing evidence relies on single-city audits, convenience samples, or administrative datasets, limiting insights into on-the-ground conditions, maintenance status, or geographic variation within states. Consequently, limited empirical evidence exists to examine co-occurring accessibility, usability, and safety patterns across diverse community contexts or to identify county-level inequities that can inform targeted planning, investment, and policy decisions.

Collectively, these gaps highlight the need for comprehensive, equity-focused empirical evaluations that examine accessibility and usability alongside playground safety as interconnected but distinct dimensions of inclusive community environments. Such evaluations are essential for determining whether parks and playgrounds function as inclusive public places that support physical activity, social participation, and belonging, and for identifying potential inequities across communities. These issues are particularly salient in Delaware, where county-level demographic differences may influence accessibility needs and inclusion priorities.

To address these gaps, this statewide cross-sectional study evaluated accessibility and usability across 50 public, non-fee-based community parks in Delaware and assessed playground safety within those parks. Building on a pilot audit of 10 Delaware parks [22], the study design and audit procedures were refined to support a comprehensive statewide assessment. Delaware provides a unique context for statewide audit due to its small geographic size, county-level governance structure, and variability in urbanicity and demographic composition, allowing for examination of accessibility patterns across diverse community contexts (primarily urban and suburban/micropolitan) within a single policy environment. Park accessibility and usability were assessed across three domains (i.e., external access, on-site amenities, communication and wayfinding) using a validated audit instrument, while playground safety was evaluated separately using a standardized playground safety tool. Descriptive analyses and exploratory comparisons by county and urbanicity category were conducted, and relationships between accessibility and safety indicators were examined to identify contextual gaps and co-occurring infrastructure limitations relevant to inclusive park use. Findings are intended to characterize publicly accessible, non-fee-based Delaware community parks with playgrounds and may not generalize to fee-based, privately operated, or rural park settings, which were not represented in the sampling frame.

Guided by the ICF [11], park participation was conceptualized as a sequential process in which external access (e.g., transit proximity, crossings, curb ramps, parking) enables arrival; on-site amenities and routes support sustained and independent use of park spaces; and communication and wayfinding features facilitate navigation and perceived inclusion. Playground safety and maintenance, assessed at the equipment level, are critical factors that can either support or deter participation in play, particularly for children with mobility, sensory, or cognitive disabilities. These domains were, therefore, examined as interconnected determinants of inclusive park use, with exploratory analyses evaluating whether fewer high-severity playground safety hazards also characterized parks with stronger accessibility and usability profiles. Accordingly, this study addressed the following research questions:

(1) What are the patterns of park accessibility and usability, and playground accessibility and safety, across public, non–fee-based parks with playgrounds in Delaware?
(2) Do park accessibility and usability, and playground accessibility and safety indicators vary by county and urbanicity category?
(3) What relationships exist between playground accessibility and life-threatening safety risks?

## 2. Materials and Methods

### 2.1. Study Design and Setting

**Figure 1:**
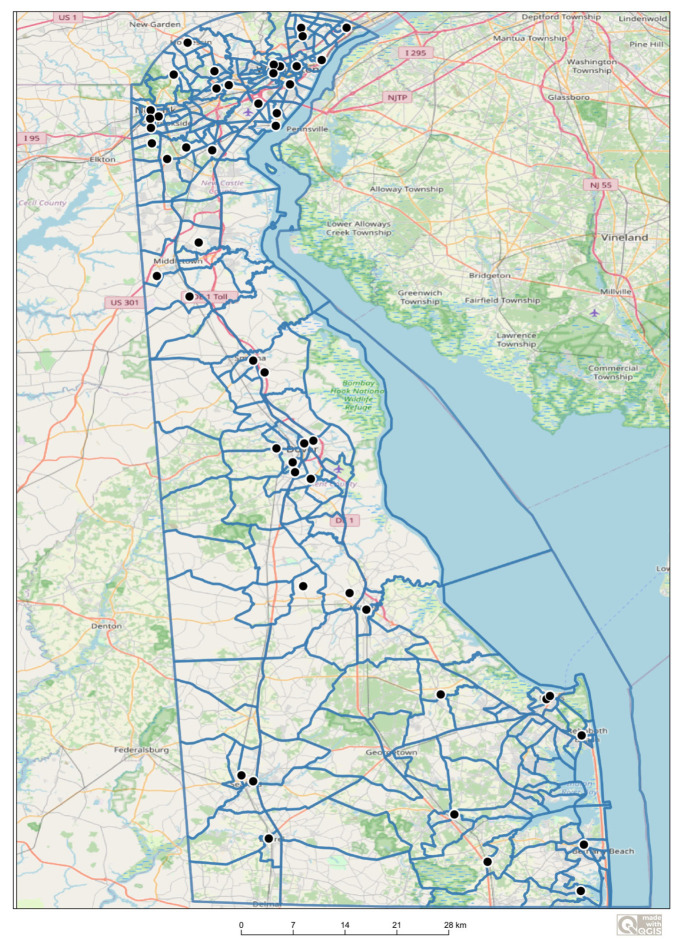
Locations of stratified Delaware community parks with playgrounds (*n* = 50). Dots represent audited park sites.

A cross-sectional observational design was utilized to collect data from a stratified sample of 50 public, non-fee-based community parks with playgrounds across Delaware’s three counties: New Castle County (NCC), Kent County (KC), and Sussex County (SC) (Figure 1). The sample size was selected to balance statewide geographic coverage with available resources and exceeded that of comparable park accessibility audits, e.g., [8]. Stratification was based on ZIP code population distributions to ensure county-level representation reflective of Delaware’s resident population; urbanicity category was not used as an a priori stratification factor.

Sampling was stratified at the county level, with the total sample (*n* = 50) allocated proportionally based on each county’s share of the state population using ZIP code–level census estimates, resulting in target samples of 28 sites in NCC (56%), 12 in SC (24%), and 10 in KC (20%). Eligible sites were identified using county and municipal park inventories and verified through map-based review. Inclusion criteria required that parks be outdoors, publicly accessible, free of entrance and parking fees, and contain fixed playground equipment intended for play and socialization. State-managed or fee-based recreation sites were excluded because they constitute a distinct park system with different access conditions and structures than those of locally managed community parks. Sites that raters deemed unsafe to assess upon arrival were also excluded (*n* = 2).

Across Delaware, 98 eligible parks with fixed playground equipment were initially identified. Within counties, eligible sites were grouped by ZIP code, assigned computer-generated random numbers using IBM SPSS Statistics, and selected in random order to reflect ZIP code population distributions while ensuring geographic coverage. From this pool, 50 sites were selected for audit; the remaining 48 eligible sites (26 in NCC, 17 in SC, 5 in KC) were not selected due to predefined sample-size constraints. Park addresses and size characteristics are provided in Appendix A, Table A1.

Urbanicity was classified post hoc using Rural-Urban Commuting Area (RUCA) codes from the U.S. Department of Agriculture [23] and the WWAMI Rural Health Research Center [24]. Following Hart et al. [25], primary RUCA codes were grouped into three categories: urban (1–3), suburban/micropolitan (4–6), and rural (7–10). Two eligible sites were located in RUCA 7 ZIP codes, both in SC; no eligible rural sites were identified in KC or NCC. Because urbanicity was not a stratification variable and rural sites were few and geographically clustered within a single county, rural–urban comparisons were not conducted. Analyses comparing urban and suburban/micropolitan contexts were therefore exploratory rather than confirmatory.

### 2.2. Instruments

Data on accessibility, usability, and safety were collected using two validated instruments. The Community Health Inclusion Index (CHII) was used to audit community recreation settings for accessibility and usability features relevant to people with disabilities [10]. The on-site form assesses external access, on-site amenities, and communication and wayfinding features, with thresholds consistent with ADAAG. Although CHII labels pedestrian ramp features as “curb cuts,” the ADAAG-preferred term “curb ramps” is used throughout this manuscript. Items include yes/no, multiple-selection, and a four-point rating scale (“none” to “all”). CHII demonstrates strong validity across 164 sites in five states, with interrater agreement ≥ 0.90 and Cronbach’s alpha values of 0.79–1.00 [10].

Playground accessibility and safety were assessed using the America’s Playgrounds Safety Report Card, adapted from the National Program for Playground Safety and aligned with the S.A.F.E.™ Playground Injury Prevention framework [26]. The tool includes 24 dichotomous items (Yes = 1, No = 0), with total scores converted to letter grades: A (20–24) to F (0–7). Twelve items with the highest injury-prevention relevance are designated as critical. Item-level playground responses were used to derive playground accessibility indicators. Critical playground safety items were used to compute a life-threatening injury risk score, as described in Section 2.4.

### 2.3. Procedures

Data were collected from June to July 2024 by three trained undergraduate research assistants. The lead rater had completed two Adapted Physical Activity courses, providing foundational knowledge of accessibility standards and disability adaptation. Prior to data collection, the first and second authors trained raters in CHII and playground safety assessment protocols. Training included at least two practice audits, and raters were required to achieve ≥95% agreement with a criterion standard before conducting independent audits.

Each rater assessed 16–17 sites over 30 days. Audits lasted approximately 60–120 min per site and included independent evaluation of accessibility, usability, and safety features. Measurements were taken using rulers and smart devices to document slope gradients, equipment height, surfacing depth/type, and interior clearances in accordance with ADAAG. Data were entered into a centralized spreadsheet along with field notes and photographs for documentation and verification.

### 2.4. Data Analysis

Datasets were reviewed by two authors for accuracy and completeness. No missing data were identified for primary variables; “not applicable” responses were excluded per instrument guidance. Data were analyzed using IBM SPSS Statistics, Version 31.0.0 (Mac), and included descriptive and exploratory inferential analyses examining county- and urbanicity-category differences and associations between accessibility and safety outcomes. Urban–suburban comparisons were considered exploratory because urbanicity was not used as a stratification factor during site selection.

Playground accessibility scores were calculated as the unweighted sum of “Yes” responses across accessibility-related playground items on the America’s Playgrounds Safety Report Card (possible range: 0–24), with higher scores indicating greater accessibility feature presence. Life-threatening injury risk scores were calculated as the sum of “No” responses across the 12 critical safety items (range: 0–12), with higher scores indicating greater injury risk. “Not applicable” responses were excluded per instrument guidance.

Descriptive statistics summarized site characteristics and domain-level scores. Dichotomous items were coded as compliant or noncompliant per ADAAG or PROWAG criteria, and results were reported as *n* (%). Ordinal coverage items (None/Some/Many/All) were summarized by category, and playground safety items were converted to letter grades per instrument scoring guidelines. Composite scores (playground accessibility and life-threatening injury risk) were summarized using median, interquartile range, and mean ± SD by county and urbanicity category (urban: RUCA 1–3; suburban/micropolitan: RUCA 4–6).

Because both composite outcomes were non-normally distributed, nonparametric tests were used. Kruskal–Wallis tests examined county-level differences, Mann–Whitney *U* tests compared urban vs. suburban/micropolitan sites, and Spearman’s rho (*ρ*) assessed associations between playground accessibility and life-threatening injury risk indices. Given unequal subgroup sizes and the exploratory aims of the study, results are interpreted primarily descriptively, and formal correction for multiple comparisons was not applied. Effect sizes were reported to support interpretation, including epsilon-squared (*ε*^2^) for Kruskal–Wallis tests and rank-biserial correlation (*r*(rb)) for Mann–Whitney *U* tests, calculated using established formulas [27]. Effect size magnitudes were interpreted using conventional nonparametric benchmarks (*ε*^2^ ≈ 0.01, 0.06, 0.14; *r*(rb) ≈ 0.10, 0.30, 0.50 for small, moderate, and large effects, respectively).

Interrater reliability was assessed through independent re-ratings of 30% of sites (agreement ≥ 0.85). Because CHII domains comprise discrete checklist items, internal consistency indices (e.g., Cronbach’s alpha) were not calculated. Consistent with prior CHII applications [10], analyses focused on rater calibration, interrater reliability, and descriptive domain-level coverage.

## 3. Results

This section presents descriptive and exploratory findings on accessibility, usability, and safety across 50 stratified Delaware parks with playgrounds (Appendix A). Results follow the visitor pathway (arrival access, on-site mobility/amenities, and playground accessibility and safety) and summarize county patterns, post hoc urban-suburban contrasts, and associations between playground accessibility and life-threatening injury risk.

### 3.1. Community and Demographic Context

The 50 audited sites were distributed across NCC (*n* = 28), KC (*n* = 10), and SC (*n* = 12), spanning 34 ZIP codes. Based on post hoc RUCA classification [23], 40 sites were in urban ZIP codes, most of which were in NCC (70.0% of urban sites), followed by KC (22.5%) and SC (7.5%). The 10 suburban/micropolitan sites were concentrated in SC (90.0%). These site-level patterns provide context for subsequent county- and setting-level comparisons.

County-level population characteristics are summarized in Table 1. According to the 2024 American Community Survey [2], disability prevalence was higher in KC and SC than in NCC, and SC had the oldest disability-age profile. Counties also differed socioeconomically, with lower median household income and higher poverty prevalence in KC and SC relative to NCC. These population-level differences contextualize observed disparities in park accessibility and safety and underscore the importance of interpreting site-level findings in relation to underlying community needs rather than as isolated environmental features.

### 3.2. Transit Stops and Arrival Access

Access to public transit at park entrances was limited statewide. Only 30% of sites (*n* = 15) had a transit stop near the entrance, with identical coverage in urban and suburban sites (30.0% each). Coverage was highest in NCC (39.3%), followed by SC (25.0%) and KC (10.0%) (Table 2). Where transit stops were present, core accessibility amenities were infrequent, including shelters (6.0%), seating (12.0%), TTY signage (14.0%), maneuverable landing space ≥ 5 ft (18.0%), lighting (20.0%), and firm landing surfaces (22.0%). These features rarely co-occurred, indicating that transit access, when available, was typically partial rather than functionally accessible. No meaningful differences were observed by urbanicity category. Notably, SC’s transit-adjacent sites consistently included seating, lighting, and firm landing surfaces, whereas KC’s single stop provided only a maneuverable landing area. These patterns suggest that the presence of transit stops alone does not ensure usable arrival access, emphasizing the importance of supporting amenities for equitable park entry.

### 3.3. Crosswalks and Intersections

Marked pedestrian crosswalks were present at 50.0% of sites, with similar coverage in urban (52.5%) and suburban (60.0%) sites. Across sites, crosswalk-related indicators were more often rated as “Some” or “Many” rather than “All,” indicating that when features were present, coverage was frequently partial rather than consistently available across approach routes (Table 3). Across all sites, 80.0% had crossings rated as obstacle-free, and 70.0% included curb ramps at both ends of the crossing, although curb-ramp coverage was lowest in KC (40.0%). Suburban sites were more likely to have curb ramps at both ends than urban sites (100% vs. 75%), though the difference was modest. In contrast, advanced pedestrian crossing aids were rare statewide, including auditory crossing signals (2.0%), visual countdown timers (8.0%), and extended crossing times accommodating slow-paced walking/rolling (8.0%). These features were most common in NCC and largely absent in KC and SC (Table 3), with minimal variation by urbanicity. Overall, crossings more often met basic structural indicators than inclusive pedestrian supports relevant to sensory, cognitive, or mobility-related access needs, indicating a gap between minimum compliance and inclusive pedestrian design.

### 3.4. Curb Ramps and Transitions

Curb ramps were needed at most sites (82.0%), but their completeness and quality were inconsistent. Among sites needing curb ramps (*n* = 41), 83.0% met ADAAG slope requirements (< 8.3%), with lower coverage in NCC (76.0%) (Table 4). Detectable warning surfaces were present at 56.1% of sites, again lower in NCC (44.0%). Most curb ramps were free of barriers or hazards (95.1%) and free of surface breaks (85.4%), with NCC showing lower coverage (76.0%). Missing curb ramps along the approach route were frequently observed (61.0%), most often in NCC (72.0%). Suburban sites more consistently met ADAAG slope requirements than urban sites (100% vs. 62.5%), but limitations related to missing curb ramps, detectable warnings, and surface condition were observed across both urbanicity categories. Overall, curb-ramp limitations reflected widespread gaps in both ramp completeness and functional quality rather than isolated site-level deficiencies.

### 3.5. Park Entry and Parking

Park entrances were largely barrier-free; only one NCC site (2.0%) had steps without an alternate route, and no sites had buildings obstructing the main entry (Table 5). Parking lots were present at 88.0% of sites (*n* = 44) and universal in SC. However, accessibility within parking areas was inconsistent. Among sites with parking, 64.0% included accessible spaces with upright signage, 54.0% had ≥5-ft access aisles, and only 32.0% had van-accessible spaces. These features showed minimal variation across counties and urbanicity categories. Together, these findings indicate that while physical entry into parks was generally unobstructed, parking-related accessibility elements that support independent arrival and transfer were frequently incomplete.

### 3.6. Pathways and Environmental Features

Pathways within parks generally supported walking and rolling (Table 6). However, several indicators were more often rated as “Some” or “Many” rather than “All,” suggesting that supports were often partial rather than consistent throughout approach routes and within parks, which may be insufficient for independent and equitable access. Most sites met basic dimensional and surface standards, including pathway width ≥ 5 ft (88.0%), obstacle-free routes (88.0%), smooth and firm surfaces (86.0%), and cross-slopes ≤ 2% (84.0%), although compliance with cross-slope requirements was lowest in KC (60.0%). Suburban sites more consistently met slope and surface standards than urban sites (100% vs. 82.5% and 80.0%, respectively).

Environmental conditions along approach routes were variable (Table 6). Clean and well-maintained sidewalks, trails, or paths were common (90.0%), with higher ratings in KC and suburban sites (both 100%). Buffers between sidewalks and streets were present at 74.0% of sites, with lower coverage in SC (50.0%). Benches or seating along streets near the entrance were available at only 50.0% of sites and were lower in SC (16.7%) and more common in urban than suburban sites (57.5% vs. 20.0%), potentially limiting opportunities for rest along approach routes. In contrast, shade trees (90.0%) and green space (94.0%) were widespread across counties.

Detracting environmental conditions were more concentrated in urban settings. Litter (70.0%), graffiti (20.0%), and loitering (42.0%) were most frequent in NCC (Table 6), whereas suburban sites more often lacked these conditions (e.g., no litter: 60.0% vs. 22.5% in urban sites; no graffiti: 100% vs. 75.0%). Noise pollution was absent at 80.0% of sites and was more commonly absent in suburban than urban sites (90.0% vs. 77.5%). Vacant buildings (8.0%) and street harassment (6.0%) were rare and occurred only in urban sites. Collectively, these patterns indicate that while core pathway infrastructure was often adequate, variation in environmental quality and supportive amenities may meaningfully influence the comfort, safety, and usability of park access, particularly for individuals with mobility, sensory, or endurance-related access needs.

### 3.7. Restrooms

Restrooms were present at 76.0% of sites (*n* = 38), with the highest availability in SC (83.3%) and the lowest in KC (70.0%) (Table 7). Availability was similar in urban (75.0%) and suburban (80.0%) sites. No restrooms had automatic doors or open corridor entrances. Low-force entry (< 5 lb) was observed at 62.0% of sites; however, fewer provided operable hardware, including lever-style door handles (32.0%) and stall latches operable with a closed fist (36.0%). Other ADAAG-aligned features were present but not universal, including door width ≥ 32 in (56.0%), grab bars (58.0%), and mobility-device-sized stalls (54.0%). Coverage of most features was highest in SC and lowest in KC, with no meaningful differences by urbanicity category, indicating that restroom availability did not consistently translate into usable access.

### 3.8. Physical Activity Areas, Playgrounds, and Multi-Use Trails

Most physical activity areas (88.0%) were at least partially accessible to mobility devices, with full accessibility observed at all KC sites and high coverage in SC (91.7%) (Table 7). Suburban sites were more likely to provide accessible activity areas than urban sites (100% vs. 85.0%). In contrast, playground accessibility was more variable. Overall, 64.0% of playgrounds had traversable surfacing and equipment with ramps or transfer platforms. SC demonstrated the highest coverage of both elevated and ground-level accessible components (83.3%), whereas KC showed the lowest coverage of traversable surfacing (40.0%) and ground-level accessible components (50.0%). Playground accessibility was higher in suburban than urban sites (80.0% vs. 60.0%).

Multi-use trails were available at 75.0% of sites (*n* = 40), with modest county variation (Table 7). Among sites with trails, 76.0% met basic width standards (≥5 ft), but fewer provided benches or rest areas (68.0%), firm and smooth surfaces (64.0%), obstacle-free routes (48.0%), or navigational aids (12.0%). Suburban sites slightly outperformed urban sites in trail surface quality (70.0% vs. 62.5%) and rest-area availability (80.0% vs. 65.0%), indicating that while trail presence was common, features supporting sustained and independent use were inconsistently available.

### 3.9. Playground Accessibility and Safety

Supervision and visibility indicators were consistently strong. Adults were present during play, and equipment was visible in 94% of playgrounds, and crawl spaces were visible in 92% (Table 8), with comparable coverage across urban (95.0%) and suburban (90.0%) playgrounds. Rules were posted at 58.0% of playgrounds, slightly higher in urban than suburban playgrounds (60.0% vs. 50.0%), while only 38.0% provided separate areas for younger and older children, with minor variation across counties and urbanicity. Age-appropriate structural design was largely compliant (guardrails: 92%; directional changes: 100%), but age-group signage was present at 64% of playgrounds, highest in KC (80%), lowest in SC (50%), and more frequent in suburban than in urban playgrounds (80.0% vs. 60.0%). Climbing-prevention features were observed in 76.0% of outer and 84.0% of internal support structures.

Surfacing and maintenance conditions were mixed. Suitable surfacing materials and covered concrete footings were nearly universal (98.0%), and six-foot use zones were present at 90.0% of playgrounds. However, only 58.0% playgrounds met loose-fill depth standards, and 70.0% met equipment height recommendations (≤8 ft), with lower compliance in NCC (57.1%). Suburban playgrounds showed slightly higher loose-fill compliance than urban playgrounds (70.0% vs. 55.0%) and higher compliance with equipment height recommendations (80.0% vs. 67.5%). Structural integrity indicators were generally high (82–100%), although rust-free equipment was less consistent (74.0%), lowest in NCC, and modestly higher in suburban vs. urban playgrounds (80.0% vs. 72.5%). Overall, 62% of playgrounds were classified as “Safe,” 28% as “On Way,” 8% as “Potentially Hazardous,” and 2% as “At Risk” (observed only in NCC).

To synthesize these patterns, composite playground accessibility and life-threatening injury risk indices were examined across counties and urbanicity categories. Higher playground accessibility scores indicate a greater number of accessibility features, whereas higher injury-risk scores indicate a greater number of absent critical safety features [26]. County-level analyses revealed no statistically significant differences in playground accessibility, *H*(2) = 5.22, *p* = 0.074, *ε*^2^ = 0.07, corresponding to a small-to-moderate effect (overall *Md* = 20.0, IQR = 18–21, *M* = 19.74 ± 2.92). Median accessibility scores were highest in KC (*Md* = 22.0, IQR = 21–22), followed by NCC (*Md* = 20.0, IQR = 18–21), and lowest in SC (*Md* = 19.5, IQR = 17–23). In contrast, county-level differences in life-threatening injury risk were statistically significant, *H*(2) = 8.65, *p* = 0.013, *ε*^2^ = 0.14, corresponding to a large effect (overall *Md* = 2.0, IQR = 1–3, *M* = 1.98 ± 1.53). Injury risk was lowest in KC (*Md* = 1.0, IQR = 0–2), followed by SC (*Md* = 2.0, IQR = 1–3), and highest in NCC (*Md* = 2.0, IQR = 1–3). Urban-suburban differences were not statistically significant for playground accessibility (*U* = 146.0, *p* = 0.187, *r*(rb) = 0.27) or life-threatening injury risk (*U* = 169.5, *p* = 0.442, *r*(rb) = 0.15); median scores were similar for urban playgrounds (accessibility: *Md* = 20.0, IQR = 18–21; injury risk: *Md* = 2.0, IQR = 1–3) and suburban playgrounds (accessibility: *Md* = 21.0, IQR = 19–22; injury risk: *Md* = 2.0, IQR = 1–3).

A strong inverse correlation was observed between playground accessibility and life-threatening safety risk (Spearman’s *ρ* = –0.80, *p* < 0.001), indicating that playgrounds with more accessibility features tended to have fewer critical safety deficiencies. Because both indices are derived from structured audit indicators and reflect overlapping aspects of design, maintenance, and management quality, this association likely reflects the co-occurrence of accessibility-oriented design and safety-supportive conditions rather than an independent causal relationship.

### 3.10. Promotional Materials

Accessibility and inclusion features in promotional materials were rare statewide (Table 7). Few sites provided accessible formats, including electronic plain text (ASCII) (8.0%), large print (12.0%), or pictograms (14.0%). Visual representation of individuals with disabilities was observed at only 6.0% of sites. Coverage was uniformly low across counties and only slightly higher in suburban than urban sites (e.g., any accessible format: 20.0% vs. 10.0%; representation of disability: 10.0% vs. 5.0%), with minimal urbanicity-category differences. These findings underscore a statewide gap in accessible and inclusive communication that may limit awareness of park features and discourage participation among individuals with disabilities, regardless of physical accessibility.

## 4. Discussion

This statewide cross-sectional audit provides the first empirical examination of accessibility, usability, and playground safety in non-fee-based parks across Delaware’s three counties, expanding on a prior northern Delaware pilot [22]. Parks and playgrounds are essential community environments that support physical activity, play, and social participation across the lifespan, yet accessibility barriers continue to disproportionately affect people with disabilities [3,5,8,30,31]. Evaluating accessibility and usability in these public settings is therefore central to identifying modifiable environmental conditions that may shape equitable opportunities for participation.

Overall, audited sites more often met basic structural indicators (e.g., ADAAG-aligned pathway design, visibility, supervision, surface conditions) than higher-level usability supports (e.g., transit-connected arrival access, curb-ramp completeness, restroom operability, navigational supports, wayfinding, trail conditions, playground usability, and accessible communication materials). Many features were frequently rated as “Some” or “Many” rather than “All,” suggesting that the feature presence did not consistently translate into continuous, independently usable access across approach routes or within parks. These gaps may constrain independent park use and limit equitable access to health-supportive community environments for people with disabilities [5,8]. Consistent with syntheses indicating that playground research often emphasizes accessibility more than usability and inclusion [20], the findings suggest uneven progress across functional domains and communities, with partial provision likely most consequential for individuals who require continuous accessible routes and predictable supports.

These findings should be interpreted as descriptive evidence of statewide strengths and gaps rather than causal determinants of physical activity or community participation. Although the observed patterns highlight actionable design and infrastructure priorities, this audit design does not allow inference that addressing gaps will directly reduce physical activity disparities. Instead, results support the need for systematic, equity-driven investment to improve the consistency and adequacy of accessibility, usability, and safety features in community parks and playgrounds, alongside future research that directly evaluates park use, participation outcomes, and user experience among people with disabilities [3,5,8,30].

### 4.1. Demographic Context

County- and urbanicity-category differences observed in the audit parallel known demographic and socioeconomic patterns across Delaware communities [2]. Because parks are place-based health resources, these environmental variations may affect equitable access to physical activity, social participation, and safety [30,31]. Urban settings in NCC, which include more racially and ethnically diverse populations [2], may benefit from culturally responsive design and, when relevant, multilingual communication strategies [30]. Because the audit did not directly assess language access or communication needs, this recommendation is based on demographic context and prior evidence. In suburban areas of SC, where the population includes a higher proportion of older adults and families with children with disabilities, age-friendly, multigenerational, and mobility-accessible features may be particularly impactful in preventing activity limitations and promoting social inclusion [17,32,33]. These contextual contrasts illustrate how demographic composition and urbanicity shape both environmental conditions and accessibility priorities [10,31]. Tailoring park and playground improvements to community-specific needs is therefore essential for advancing public health equity and creating health-supportive environments that foster participation, inclusion, and social connection [3,5].

### 4.2. Foundational Accessibility

Foundational accessibility refers to structural features (e.g., pedestrian crossings, curb ramps, accessible parking, transit stops) that enable people to reach parks and move safely within and around them [9,10,13]. These features are important built-environment conditions that influence whether individuals, particularly people with disabilities and households without vehicles, can reliably access community environments that support physical activity and social participation [3,5,31]. Despite approximately 26,000 Delaware households lacking access to a vehicle [2], only 30% of audited parks had a nearby transit stop, with lower coverage in KC and SC. Where stops were present, key accessibility features, including shelters, seating, and firm/maneuverable landing areas, were uncommon and rarely co-occurred. These findings mirror prior reports of limited stop proximity, inconsistent design, and weak coordination between transportation and parks/recreation agencies [10,22,34]. Additional qualitative evidence highlights system-level constraints (e.g., limited accessible vehicles, long waits, sparse rural coverage) and built-environment barriers (e.g., uneven surfaces, steep slopes) that undermine the usability of transit for people with disabilities [16,34].

Pedestrian infrastructure showed similar gaps. Marked crosswalks were present at only half of the sites, and although curb ramps were common, detectable warning surfaces were frequently missing, and curb-ramp completeness was inconsistent across approach routes. SC, which included more suburban/micropolitan sites, outperformed NCC and KC on several curb-ramp condition indicators, whereas NCC had the highest prevalence of missing curb ramps along the approach route. Suburban parks had somewhat better ramp coverage but not consistently higher quality, indicating that compliance challenges occur across both urban and suburban settings. These patterns reflect national trends in which pedestrian upgrades are incremental and lack routine maintenance [8,13,33,35]. Advanced pedestrian aids, particularly accessible pedestrian signals (APS), were rarely observed and largely absent in KC and SC, consistent with low national APS deployment [13,36]. Their absence may limit safe and independent navigation for individuals who rely on visual, tactile, and temporal cues [13,33], contributing to preventable mobility-related inequities. Federal guidance reinforces this need: PROWAG requires APS at pedestrian signal locations [13], and the Manual on Uniform Traffic Control Devices recommends countdown timers and extended signal phases to accommodate slower walking speeds and wheelchair users [35].

Parking infrastructure was more consistently available statewide but frequently lacked upright signage, adequately wide aisles, or van-accessible spaces, limitations echoed in other park and trail audits [8,22]. In contrast, entry-related barriers were rare, reflecting the generally open design of many Delaware settings. Collectively, these foundational accessibility gaps represent modifiable built-environment conditions that may constrain community mobility and safe park access, reinforcing the need for coordinated, cross-agency investment to create a more equitable and inclusive community environment [3,5,31,37]. From an implementation perspective, patterns observed here suggest that KC may benefit from improving transit stop availability and supporting amenities, whereas NCC may warrant greater attention to curb-ramp completeness and approach-route continuity.

### 4.3. Amenities, Usability, and Safety

Amenities and usability influence whether visitors can participate safely, comfortably, and inclusively once inside a park [38]. Consistent with prior audits [8,22], the provision of these features was uneven across Delaware’s parks. This pattern suggests that functional access depends not only on whether features exist, but also on whether they are consistently available to support independent use. For example, in a multi-city study, each one-point increase in composite park quality increased the odds of use by 2%, with a stronger association for women [38]. Conversely, gaps in benches, rest areas, or pathway conditions can undermine comfort and limit usability [8,22].

Primary pathways generally met ADAAG standards for width, slope, and surface firmness, supporting basic walkability and rollability. However, supportive amenities, including benches and buffers between pathways and streets, were inconsistently available, particularly benches in SC. Shade trees and green space were common, but detracting conditions (litter, graffiti, loitering) were more prevalent in NCC’s urban parks, aligning with research linking physical disorder to reduced perceived safety and park use [30,31]. These patterns support conceptual models and prior audits demonstrating that amenities, environmental quality, and safety cues shape park comfort, usability, and inclusivity [10,22,31].

Multi-use trails showed greater variability than primary pathways. Although most met the five-foot width standard, fewer had consistently smooth surfaces, obstacle-free routes, or navigational aids. Qualitative studies highlight how uneven surfaces, steep grades, and limited navigational aids hinder independent navigation for users with mobility limitations [16]. KC outperformed NCC and SC on surface quality and obstacle-free routes, though no county consistently met all criteria. These patterns indicate that trails may meet basic dimensional standards (e.g., width) yet fall short on practical usability for many users, particularly individuals with mobility disabilities, consistent with research documenting how built-environment conditions constrain safe and independent movement [16,31]. At the systems level, these gaps also reflect national challenges in maintaining extensive park and trail networks, with many agencies reporting substantial deferred maintenance burdens [39]. These findings reinforce the need for equity-focused reinvestment in trail infrastructure to enhance accessibility, usability, and opportunities for active transportation [8,10].

Restroom availability was relatively high (76%), but accessibility features essential for independent use, such as automatic doors, low-force entry, operable hardware, and adequate stall space, were frequently missing. Across restroom features assessed, coverage was highest in SC and lowest in KC, consistent with other audits documenting similar restroom-related barriers [8,10,16,22]. Such barriers are often described by individuals with disabilities as humiliating or exclusionary [16], underscoring the need for user-centered restroom design that supports dignity, autonomy, and independence.

Physical activity areas were often partially accessible, with near-universal access in KC and high access in SC, and suburban parks were more likely to provide accessible facilities than urban parks. Playground accessibility showed a similar pattern: about two-thirds of playgrounds had traversable surfacing, ground-level components, or ramped/transfer-accessible structures, consistent with prior audits [8,22]. SC demonstrated the strongest coverage of ramped/transfer-accessible structures and ground-level components, whereas KC had the lowest rates of traversable surfaces and ground-level components. Although these contributing factors were not directly measured, observed county patterns may reflect differences in infrastructure condition, maintenance capacity, and local investment context. Socioeconomic context may also contribute; KC’s lower median household income [2] may limit the pace of accessibility upgrades, consistent with evidence that parks in higher-income areas tend to receive greater investment [30,39]. Targeted accessibility improvements may therefore be needed to enhance park quality—an important predictor of park use [38].

Playground safety generally met national benchmarks, with strong compliance in supervision, visibility, and structural features associated with reduced injury risk. However, consistent with prior audits [21], gaps persisted in signage, loose-fill depth, equipment height, and age-appropriate design. Fewer than half of playgrounds provided separated areas for younger and older children, and compliance with loose-fill and equipment-height standards was inconsistent, particularly in NCC, where taller structures and deferred maintenance were more common. Although most playgrounds were classified as “Safe,” approximately 10% were rated as “Potentially Hazardous” or “At Risk,” slightly lower than the 17% reported by Suminski et al. [21]. KC demonstrated the strongest accessibility and safety profiles, whereas NCC showed comparatively lower performance despite its larger, more urban park system. The urbanicity category was not associated with accessibility or injury risk, suggesting that the observed variation reflects local design, maintenance, and investment practices rather than the urban-suburban context.

A strong inverse association was observed between playground accessibility and risk of life-threatening injury; however, this relationship should be interpreted descriptively rather than causally. Both indices reflect overlapping aspects of design quality, maintenance, and management attention, and the observed correlation likely represents the co-occurrence of accessibility-oriented and safety-supportive conditions rather than an independent effect of accessibility features on injury risk, consistent with prior playground audit findings [21]. While causal links to physical activity or injury outcomes cannot be inferred from this cross-sectional audit, these findings support the potential value of integrated, equity-focused reinvestment strategies that pair accessibility upgrades with routine maintenance and equitable capital planning [39].

### 4.4. Communication and Wayfinding

Communication and wayfinding shape how individuals learn about, navigate, and feel represented in parks. Within the ICF, communication supports and information systems and services are environmental factors that enable activity and participation; when they are absent or inaccessible, activities may be constrained even where physical assets exist [11]. In this audit, accessible communication formats were rare. Only a small proportion of parks provided accessible formats (e.g., electronic plain text, large print, pictograms) or any visual representation of people with disabilities. These gaps restrict access to information and may also limit the extent to which parks convey inclusivity or belonging, both of which are central components of effective public messaging [40,41]. Urbanicity-category differences were minimal, suggesting these limitations are statewide rather than localized. Qualitative evidence highlights several practical solutions, including legible, well-placed information boards; maps that clearly identify accessible routes and restrooms; and the use of Braille and other non-text formats along pathways [16]. Co-developing content with people with disabilities can move participation beyond tokenism, where voice is present but decision-making power is limited, toward meaningful shared design [42]. Expanding accessible formats and incorporating authentic, diverse imagery through collaborative design are consistent with universal design and inclusion recommendations for communication supports within park settings, including larger font sizes, multilingual materials, and web-accessible content [5,39].

### 4.5. Strengths and Limitations

Although this study used validated instruments, a stratified statewide sample (by county and ZIP-code population distribution), and trained raters with strong inter-rater agreement, several limitations should be considered. First, findings from a single state may not be generalizable to regions with different demographics, infrastructure conditions, climates, or policy environments. Second, although urbanicity was categorized using RUCA codes, it was not used as a stratification variable during site selection. Only two eligible sites were located in rural (RUCA 7) ZIP codes, both in SC, which limited examination of accessibility patterns across the full urban–rural continuum. Accordingly, urbanicity-based comparisons are interpreted as exploratory and descriptive rather than confirmatory. Third, excluding fee-based or privately operated settings narrows representativeness and may underestimate disparities across different recreation systems. Fourth, observational audits capture objective features but not lived experience, perceived safety, or social dynamics, all of which shape inclusivity. Daytime data collection may also overestimate cleanliness, supervision, or perceived safety relative to evening or weekend conditions. Finally, field measurements and observations may introduce minor errors despite strong inter-rater reliability.

Future studies should integrate mixed methods, including interviews or co-audits with people with disabilities; expand to rural and fee-based settings; and examine longitudinal changes to assess how upgrades, maintenance, and programming influence participation and health outcomes.

### 4.6. Implications for Research and Practice

For research, statewide audits that use standardized measures of accessibility, usability, and safety provide a starting point for monitoring equity-related differences across community environments. These audits also help assess whether people with disabilities can expect a consistent, high-quality experience across sites, an area that warrants more investigation. Future studies should examine the perspectives of park and playground users, especially people with disabilities, to combine observational data with lived experience. Research is also needed to evaluate whether improvements in infrastructure, communication, and programming are associated with changes in park use, physical activity, and related outcomes. Comparative analyses across counties or states may help identify effective strategies and guide policy.

For practice, ADAAG compliance represents a minimum threshold and does not ensure meaningful or independent participation [10,43]. Public health agencies, local health departments, and parks and recreation providers should implement routine monitoring, prioritize accessibility upgrades, and apply universal design principles that anticipate diverse user needs [14]. Evidence-based frameworks such as the Guidelines, Recommendations, and Adaptations Including Disability (GRAIDs) [5] offer step-by-step guidance for adapting existing programs and facilities to support disability-inclusive participation. Based on the gaps observed in this audit, priority areas include accessible restrooms, available and reasonably spaced benches or resting points, clear wayfinding supports, and developmentally appropriate play structures. County-level variation observed in this study suggests that improvements should be tailored to local demographic and environmental contexts rather than adopting a uniform model. For example, KC and SC may benefit from improvements in transit access (e.g., stop availability/proximity and supporting amenities) and pedestrian crossing infrastructure (e.g., marked crossings and curb ramps at both ends of crossings). In contrast, NCC may warrant added attention to curb-ramp completeness and environmental conditions near entrances, and SC may also benefit from more benches/rest points to support sustained use.

Treating accessibility as a public health priority underscores the role of inclusive parks in supporting daily activity and well-being. Inclusive park and playground design may expand physical activity opportunities for groups that have historically faced greater barriers, aligning with international recommendations for coordinated action to improve equitable access to outdoor recreation [44]. Routine audits can help monitor progress, but meaningful inclusion also requires co-developing priorities and materials with people with disabilities to move participation beyond tokenism toward shared decision-making [42]. Integrating legal standards, universal design, and participatory practices can help ensure that parks and playground environments support physical activity, social participation, and belonging for all.

## 5. Conclusions

Many Delaware parks with playgrounds met foundational accessibility and safety benchmarks, particularly in pathway design and key playground safety indicators such as visibility and supervision. However, substantial gaps were evident in transit access, pedestrian crossings, curb ramps, parking, restrooms, trail usability, and inclusive play features. Communication and wayfinding supports were limited, suggesting that many settings may lack clear and accessible information for visitors with diverse needs. County-level differences highlighted uneven progress across local environments, whereas urbanicity (urban vs. suburban/micropolitan) was not associated with accessibility or injury risk. Interpreted through the ICF framework, these findings show that independent use of parks and playgrounds depends not only on individual features but on how multiple environmental elements function together to facilitate or hinder participation [11]. Consistent with prior research, compliance with ADAAG and PROWAG represents a minimum threshold; meaningful and sustained use also depends on supportive amenities, reliable maintenance, clear and accessible information, and environments that promote physical comfort, safety, and belonging [16,37]. Ensuring that community parks and playgrounds function as health-supportive places will therefore require continued attention to these interconnected dimensions of accessibility. Positioned within a public health and health equity framework, these findings can inform planning, transportation, and parks and recreation policies to address environmental barriers that may contribute to disparities in physical activity and community participation among people with disabilities in Delaware and similar jurisdictions.

## Figures and Tables

**Table 1:** Demographic Characteristics of Delaware and Counties, American Community Survey 2024 (1-Year Estimates).

Population Characteristic	Delaware (*n* = 50)	NCC (*n* = 28)	KC (*n* = 10)	SC (*n* = 12)
Total Population, *N* (% with disability)	1,037,354 ± 1568 (14.0 ± 0.8)	580,531 ± 655 (12.5 ± 1.1)	188,555 ± 1199 (16.8 ± 2.0)	268,268 ± 549 (15.2 ± 1.4)
Median Age (years)	41.5 ± 0.1	38.5 ± 0.4	39.2 ± 0.2	51.4 ± 0.2
Disability by Age Group, N (years)				
Under 5 years	545 ± 445	506 ± 433	0 ± 208	39 ± 59
5 to 17 years	9766 ± 2480	4503 ± 1485	1645 ± 792	3618 ± 1655
18 to 34 years	19,924 ± 2974	11,605 ± 2244	4537 ± 1369	3782 ± 1655
35 to 64 years	46,886 ± 4256	24,208 ± 3540	11,758 ± 2182	10,920 ± 1967
65 to 74 years	29,875 ± 3161	13,117 ± 2209	6864 ± 1356	9894 ± 1422
75+ years	38,130 ± 2175	18,807 ± 1372	6858 ± 996	12,465 ± 1325
Sex, N (% with disability)				
Female	540,395 ± 1595 (14.1 ± 0.9)	300,177 ± 897 (13.0 ± 1.2)	99,642 ± 565 (16.7 ± 2.4)	140,576 ± 1201 (14.6 ± 1.7)
Male	496,959 ± 1927 (13.9 ± 1.0)	280,354 ± 576 (12.0 ± 1.4)	88,913 ± 1206 (16.9 ± 2.4)	127,692 ± 1459 (15.9 ± 1.8)
Race/Ethnicity, N (% with disability)				
Asian alone ^a^	50,727 ± 1570 (7.0 ± 1.8)	40,467 ± 1472 (5.8 ± 1.7)	5931 ± 400 (14.8 ± 9.0)	4329 ± 261 (7.2 ± 7.7)
Black/African American alone ^a^	226,593 ± 6735 (13.6 ± 1.9)	153,206 ± 3960 (12.9 ± 2.4)	47,788 ± 3723 (16.2 ± 4.5)	25,599 ± 3230 (13.0 ± 4.1)
White alone ^a^	604,435 ± 6203 (15.0 ± 0.7)	299,438 ± 4514 (13.5 ± 1.1)	105,771 ± 1845 (18.0 ± 2.3)	199,226 ± 3336 (15.6 ± 1.5)
Two or More Races	94,094 ± 7943 (12.7 ± 2.5)	51,368 ± 6424 (11.8 ± 3.0)	22,315 ± 4216 (15.1 ± 5.5)	20,411 ± 4345 (12.5 ± 4.7)
Hispanic/Latino (any race)	121,353 ± 734 (9.7 ± 2.4)	72,884 ± 436 (10.1 ± 3.1)	15,798 ± 544 (5.7 ± 2.8)	32,671 ± 118 (11.0 ± 5.1)
Household Income & Poverty				
Median (USD)	82,855 ± 1234	89,901 ± 1993	72,872 ± 1932	78,162 ± 1865
Mean (USD)	109,519 ± 1313	118,003 ± 1977	89,176 ± 2271	105,000 ± 2259
% Below Poverty	10.7 ± 0.5	10.2 ± 0.5	11.3 ± 1.0	11.5 ± 1.0

Note. Values are estimates ± margins of error (90% CI). NCC = New Castle County; KC = Kent County; SC = Sussex County. Median and mean household income values are from the U.S. Census Bureau, ACS 2024 1-year, Table S1901 [28]. Poverty data are from the 2024 1-year ACS, Table S1701 [29]. Race/ethnicity categories marked with “^a^” are non-Hispanic.

**Table 2:** Transit Stop Features Near Park Entrances.

Accessibility Feature	Delaware (*n* = 50)	NCC (*n* = 28)	KC (*n* = 10)	SC (*n* = 12)
Transit stops near the site entrance	15 (30.0)	11 (39.3)	1 (10.0)	3 (25.0)
Shelter at the transit stop	3 (6.0)	3 (10.7)	0 (0.0)	0 (0.0)
Seating at the transit stop	6 (12.0)	3 (10.7)	0 (0.0)	3 (25.0)
TTY signage at the transit stop	7 (14.0)	5 (17.9)	0 (0.0)	2 (16.7)
Space for mobility device maneuvering (≥5 ft)	9 (18.0)	7 (25.0)	1 (10.0)	1 (8.3)
Stable, firm landing surface at transit stop	11 (22.0)	7 (25.0)	1 (10.0)	3 (25.0)
Lighting at or near the transit stop	10 (20.0)	7 (25.0)	0 (0.0)	3 (25.0)

Note. Values are frequency (%). NCC = New Castle County, KC = Kent County, SC = Sussex County.

**Table 3:** Crosswalk Accessibility Features Near Park Entrances.

Accessibility Feature	Delaware (*n* = 50)			NCC (*n* = 28)	KC (*n* = 10)	SC (*n* = 12)
	All	Many	Some	Any Presence *		
Crosswalk marked with stripes/paint/bricks	11 (22.0)	7 (14.0)	7 (14.0)	15 (53.6)	5 (50.0)	5 (41.7)
Crossing free of obstacles/hazards	22 (44.0)	13 (26.0)	5 (10.0)	24 (85.7)	7 (70.0)	9 (75.0)
Curb ramps at both ends of the crossing	25 (50.0)	7 (14.0)	3 (6.0)	22 (78.6)	4 (40.0)	9 (75.0)
Auditory crossing signals near the site	0 (0.0)	1 (2.0)	0 (0.0)	0 (0.0)	1 (10.0)	0 (0.0)
Visual countdown timers at traffic signals near the site	2 (4.0)	2 (4.0)	0 (0.0)	3 (10.7)	1 (10.0)	0 (0.0)
Traffic signal crossing time accommodates slow-paced walking/rolling	2 (4.0)	2 (4.0)	0 (0.0)	3 (10.7)	1 (10.0)	0 (0.0)

Note. Values are frequency (%). NCC = New Castle County, KC = Kent County, SC = Sussex County. * For county columns, values represent any presence (All/Many/Some combined). Delaware columns report All/Many/Some separately.

**Table 4:** Curb-Ramp Condition Features on Approach Near Park Entrances.

Accessibility Feature	Delaware (*n* = 50)			NCC (*n* = 28)	KC (*n* = 10)	SC (*n* = 12)
Curb ramps are needed on the approach route (Yes)	41 (82.0)			25 (89.3)	5 (50.0)	11 (91.7)
Among sites where curb ramps were needed:						
Accessibility Feature	All ^a^	Many ^a^	Some ^a^	Any presence *^,a^		
	(*n* = 41)	(*n* = 41)	(*n* = 41)	(*n* = 25)	(*n* = 5)	(*n* = 11)
Curb-ramp slope is <8.3%	27 (65.9)	5 (12.2)	2 (4.9)	19 (76.0)	5 (100)	10 (90.9)
Curb ramps free of barriers or hazards	25 (61.0)	11 (26.8)	3 (7.3)	23 (92.0)	5 (100)	11 (100)
Curb-ramp surface free of breaks	29 (70.7)	2 (4.9)	4 (9.8)	19 (76.0)	5 (100)	11 (100)
Detectable warning surface in good condition	11 (26.8)	5 (12.2)	7 (17.1)	11 (44.0)	4 (80.0)	8 (72.7)
No curb ramps present	5 (12.2)	5 (12.2)	15 (36.6)	18 (72.0)	3 (60.0)	4 (36.4)

Note. Values are frequency (%). NCC = New Castle County, KC = Kent County, SC = Sussex County. CHII uses ‘curb cuts,’ reported here as ‘curb ramps’ following ADAAG. * For county columns, values represent any presence (All/Many/Some combined). Delaware columns report All/Many/Some separately. ^a^ Percentages for curb-ramp condition items are calculated among sites where curb ramps were needed (Delaware *n* = 41; NCC *n* = 25; KC *n* = 5; SC *n* = 11).

**Table 5:** Parking Accessibility Features Within Parks.

Accessibility Feature	Delaware (*n* = 50)	NCC (*n* = 28)	KC (*n* = 10)	SC (*n* = 12)
Parking lot available at the park	44 (88.0)	23 (82.1)	9 (90.0)	12 (100)
Accessible spaces designated with the International Symbol of Accessibility on an upright sign in the lot (min. 60″)	32 (64.0)	16 (57.1)	7 (70.0)	9 (75.0)
Accessible parking aisles (≥5 ft)	27 (54.0)	13 (46.4)	6 (60.0)	8 (66.7)
Designated van-accessible spaces	16 (32.0)	9 (32.1)	3 (30.0)	4 (33.3)

Note. Values are frequency (%). NCC = New Castle County, KC = Kent County, SC = Sussex County.

**Table 6:** Pathway and Environmental Accessibility Features Within Parks.

Accessibility Feature	Delaware (*n* = 50)			NCC (*n* = 28)	KC (*n* = 10)	SC (*n* = 12)
	All	Many	Some	Any Presence *		
Clean/well-maintained sidewalks, trails, or paths	14 (28.0)	23 (46.0)	8 (16.0)	25 (89.3)	10 (100)	10 (83.3)
Buffer between sidewalk and street	15 (30.0)	11 (22.0)	11 (22.0)	22 (78.6)	9 (90.0)	6 (50.0)
Benches or seating along streets near the entrance	0 (0.0)	8 (16.0)	17 (34.0)	16 (57.1)	7 (70.0)	2 (16.7)
Trees/shade along streets near the entrance	7 (14.0)	17 (34.0)	21 (42.0)	26 (92.9)	10 (100)	9 (75.0)
Green space along streets near the entrance	12 (24.0)	25 (50.0)	10 (20.0)	26 (92.9)	10 (100)	11 (91.7)
Free of noise pollution	8 (16.0)	18 (36.0)	14 (28.0)	21 (75.0)	10 (100)	9 (75.0)
People loitering around the site present	0 (0)	1 (2.0)	20 (40.0)	14 (50.0)	4 (40.0)	3 (25.0)
Graffiti around the site present	0 (0)	2 (4.0)	8 (16.0)	9 (32.1)	1 (10.0)	0 (0.0)
Littering around the site present	1 (2.0)	7 (14.0)	27 (54.0)	25 (89.3)	6 (60.0)	4 (33.3)
Vacant buildings around the site present	0 (0)	0 (0)	4 (8.0)	3 (10.7)	0 (0.0)	1 (8.3)
Street harassment around the site present	0 (0)	0 (0)	3 (6.0)	2 (7.1)	0 (0.0)	1 (8.3)
Pathways ≥ 5 feet wide	24 (48.0)	12 (24.0)	8 (16.0)	24 (85.7)	10 (100)	10 (83.3)
Path free of obstacles or hazards	12 (24.0)	18 (36.0)	14 (28.0)	24 (85.7)	9 (90.0)	11 (91.7)
Path cross slope ≤ 2% (1.1°)	33 (66.0)	5 (10.0)	4 (8.0)	25 (89.3)	6 (60.0)	11 (91.7)
Path surface smooth/firm (no gravel/dirt)	29 (58.0)	10 (20.0)	4 (8.0)	24 (85.7)	9 (90.0)	10 (83.3)

Note. Values are frequency (%). NCC = New Castle County, KC = Kent County, SC = Sussex County. * For county columns, values represent any presence (All/Many/Some combined). Delaware columns report All/Many/Some separately.

**Table 7:** Accessibility Features of Restrooms, Physical Activity Areas, Playgrounds, Multi-Use Trails, and Promotional Materials Within Parks.

Accessibility Feature	Delaware (*n* = 50)	NCC (*n* = 28)	KC (*n* = 10)	SC (*n* = 12)
Restrooms				
Restroom present	38 (76.0)	21 (75.0)	7 (70.0)	10 (83.3)
Automatic door or open corridor entrance	0 (0.0)	0 (0.0)	0 (0.0)	0 (0.0)
Minimal force (<5 lb) to open the restroom door	31 (62.0)	17 (60.7)	4 (40.0)	10 (83.3)
Door handles operable with a closed fist	16 (32.0)	8 (28.6)	3 (30.0)	5 (41.7)
Door opening ≥32″ wide	28 (56.0)	17 (60.7)	4 (40.0)	7 (58.3)
Stall handles/latches operable with a closed fist	18 (36)	11 (39.3)	2 (20.0)	5 (41.7)
Grab bars in the stall	29 (58)	17 (60.7)	5 (50.0)	7 (58.3)
Stall space for mobility device (60″ × 56–59″)	27 (54.0)	16 (57.1)	4 (40.0)	7 (58.3)
Physical Activity Areas				
Spaces within physical activity areas accessible for mobility devices	44 (88.0)	23 (82.1)	10 (100)	11 (91.7)
All	13 (26.0)	7 (25.0)	2 (20.0)	4 (33.3)
Many	12 (24)	5 (17.9)	4 (40.0)	3 (25.0)
Some	19 (38.0)	11 (39.3)	4 (40.0)	4 (33.3)
Playgrounds				
Ground material traversable by a mobility device	32 (64.0)	20 (71.4)	4 (40.0)	8 (66.7)
Elevated equipment with ramps or transfer platforms	30 (60.0)	14 (50.0)	6 (60.0)	10 (83.3)
Ground-level play components usable by a person using a mobility device	31 (62.0)	16 (57.1)	5 (50.0)	10 (83.3)
Multi-Use Trails				
Benches or rest areas along the trail	34 (68.0)	20 (71.4)	7 (70.0)	7 (58.3)
Firm, smooth surface on the trail	32 (64.0)	17 (60.7)	8 (80.0)	7 (58.3)
Trail width ≥5 ft for mobility devices	38 (76.0)	21 (75.0)	9 (90.0)	8 (66.7)
Trail free of obstacles or hazards	24 (48.0)	12 (42.9)	7 (70.0)	5 (41.7)
Trail has navigational aids	6 (12.0)	3 (10.7)	1 (10.0)	2 (16.7)
Promotional Materials				
Electronic plain text (ASCII)	4 (8.0)	3 (10.7)	1 (10.0)	0 (0.0)
Large print	6 (12.0)	4 (14.3)	1 (10.0)	1 (8.3)
Pictograms	7 (14.0)	5 (17.9)	1 (10.0)	1 (8.3)
Inclusion of persons with disabilities	3 (6.0)	2 (7.1)	0 (0.0)	1 (8.3)

Note. Values are frequency (%). NCC = New Castle County, KC = Kent County, SC = Sussex County.

**Table 8:** Playground Accessibility and Safety Features Based on America’s Playground Safety Report Card.

Safety Feature	Delaware (*n* = 50)	NCC (*n* = 28)	KC (*n* = 10)	SC (*n* = 12)
Supervision				
Adults are present when children use the equipment	47 (94.0)	26 (92.9)	9 (90.0)	12 (100)
Children are easily viewed on the equipment	47 (94.0)	27 (96.4)	9 (90.0)	11 (91.7)
Children are easily viewed in crawl spaces	46 (92.0)	26 (92.9)	10 (100)	10 (83.3)
Rules posted regarding expected behavior	29 (58.0)	15 (53.6)	6 (60.0)	8 (66.7)
Age-Appropriate Design				
Separate areas for ages 2–5 years and 5–12 years	19 (38.0)	10 (35.7)	6 (60.0)	3 (25.0)
Platforms have appropriate guardrails	46 (92.0)	25 (89.3)	10 (100)	11 (91.7)
Platforms allow a change in direction to get on/off the structure	50 (100)	28 (100)	10 (100)	12 (100)
Signage indicating the age group for the equipment provided	32 (64.0)	18 (64.3)	8 (80.0)	6 (50.0)
Equipment design prevents climbing outside the structure	38 (76.0)	22 (78.6)	8 (80.0)	8 (66.7)
Supporting structures prevent climbing on them	42 (84.0)	22 (78.6)	9 (90.0)	11 (91.7)
Fall Surfacing				
Suitable surfacing materials provided	49 (98.0)	28 (100)	10 (100)	11 (91.7)
Height of equipment is ≤8 ft	35 (70.0)	16 (57.1)	8 (80.0)	11 (91.7)
Appropriate depth of loose fill provided	29 (58.0)	15 (53.6)	9 (90.0)	5 (41.7)
The 6-foot use zone has appropriate surfacing	45 (90.0)	23 (82.1)	10 (100)	12 (100)
Concrete footings are covered	49 (98.0)	27 (96.4)	10 (100)	12 (100)
Surface free of foreign objects	27 (54.0)	14 (50.0)	6 (60.0)	7 (58.3)
Equipment Maintenance				
Equipment free of noticeable gaps	48 (96.0)	26 (92.9)	10 (100)	12 (100)
Equipment free of head entrapments	45 (90.0)	23 (82.1)	10 (100)	12 (100)
Equipment free of broken parts	42 (84.0)	23 (82.1)	9 (90.0)	10 (83.3)
Equipment free of missing parts	47 (94.0)	26 (92.9)	10 (100)	11 (91.7)
Equipment free of protruding bolts	48 (96.0)	26 (92.9)	10 (100)	12 (100)
Equipment is free of rust	37 (74.0)	19 (67.9)	8 (80.0)	10 (83.3)
Equipment is free of splinters	47 (94.0)	27 (96.4)	9 (90.0)	11 (91.7)
Equipment is free of cracks/holes	43 (86.0)	23 (82.1)	10 (100)	10 (83.3)
Mean Playground Accessibility ^a^	19.74 ± 2.92	19.11 ± 3.19	21.40 ± 1.51	19.83 ± 2.76
Mean Life-Threatening Injury Risk ^b^	1.98 ± 1.53	2.43 ± 1.64	0.90 ± 0.88	1.83 ± 1.27
Risk Classification: Safe	31 (62)	16 (57.1)	9 (90.0)	6 (50.0)
Risk Classification: On Way	14 (28)	8 (28.6)	1 (10.0)	5 (41.7)
Risk Classification: Potentially Hazardous	4 (8)	3 (10.7)	0 (0.0)	1 (8.3)
Risk Classification: At Risk	1 (2)	1 (3.6)	0 (0.0)	0 (0.0)

Note. Values are frequency (%) unless indicated otherwise. NCC = New Castle County, KC = Kent County, SC = Sussex County. Urbanicity category comparisons (urban, RUCA 1–3; suburban/micropolitan, RUCA 4–6) were conducted only for composite accessibility and safety indices. ^a^ Playground accessibility score represents the unweighted sum of “Yes” responses across accessibility-related playground items. ^b^ Life-threatening injury risk score represents the sum of missing (No) responses across the 12 critical safety items.

## Data Availability

The data supporting this study are available from the corresponding author upon reasonable request for non-commercial research purposes.

## Abbreviations

The following abbreviations are used in this manuscript:

ADA	Americans with Disabilities Act
ADAAG	Americans with Disabilities Act Accessibility Guidelines
APS	Accessible pedestrian signals
CHII	Community Health Inclusion Index
ICF	International Classification of Functioning, Disability, and Health
KC	Kent County
NCC	New Castle County
PROWAG	Public Rights-of-Way Accessibility Guidelines
RUCA	Rural-Urban Commuting Area Codes
S.A.F.E.™	Supervision, Age-appropriate design, Fall surfacing, Equipment maintenance
SC	Sussex County

## Appendix A

**Table A1:** Characteristics of the Parks with the Included Playgrounds.

ID	Park Name (Inaugural Year)	County	Size (Acres)	Physical Activity-Related Amenities	Address
1	Kings Croft Park (1994)	NCC	7.5	Baseball/Softball Field, Multipurpose Fields, PG, Trails/Paths	1 Regal Blvd, Bear, DE 19701
2	Glasgow Regional Park (NR; 2016 for H!gh 5 Sensory PG)	NCC	250	Basketball Courts, H!gh 5 Sensory PG ^†^, PG, Tennis Courts, Trails/Paths	2275 Pulaski Hwy, Newark, DE 19702
3	Iron Hill Park (1968)	NCC	335	Disc Golf Course, Multipurpose Fields, PG, Trails/Paths	1337 S Old Baltimore Pike, Newark, DE 19702
4	Becks Pond Park (NR)	NCC	28	PG	793 Salem Church Road, Newark, DE 19702
5	Woods Haven Kruse Park (NR)	NCC	44.9	Multipurpose Fields, PG, Trails/Paths	100 Darley Rd, Claymont, DE 19703
6	Swift Memorial Park (1976)	NCC	30	Baseball Field, PG, Soccer Field, Trails/Paths	1001 Valley Rd Hockessin, DE 19707
7	Charles Price Memorial Park (2007)	NCC	100	Multipurpose Fields, PG, Trails/Paths	955 Levels Rd, Middletown, DE 19709
8	NCC Southern Park (2023)	NCC	100	Baseball/Softball Field, Basketball Courts, Fitness Equipment, Gaga Pit, Multipurpose Fields, Pickleball Courts, PG, Recreation Programs, Tennis Courts, Trails/Paths, Zipline	1276 Shallcross Lake Rd, Middletown, DE 19709
9	Kells Park (NR)	NCC	5.2	Baseball/Softball Field, Basketball Courts, PG, Soccer Field, Tennis Practice Wall, Trails/Paths	201 Kells Ave, Newark, DE 19711
10	Paper Mill Park (2008)	NCC	29.3	Basketball Courts, PG, Soccer Field, Tennis Courts, Trails/Paths	1050 Paper Mill Rd, Newark, DE 19711
11	Phillips Park (1974)	NCC	13.7	Basketball Courts, PG, Skate Park, Tennis Courts, Trails/Paths	101 B Street, Newark DE 19711
12	Hillside Park (2021)	NCC	7	Natural Play Area, PG, Trails/Paths	151 Forest Lane, Newark, DE 19711
13	Rittenhouse Park (1974)	NCC	45.9	PG, Trails/Paths	228 W Chestnut Hill Rd, Newark, DE 19713
14	Chelsea Manor Park (2005)	NCC	10.6	Baseball/Softball Field, PG, Soccer Field, Trails/Paths	0 West Roosevelt Avenue, New Castle DE 19720
15	New Castle Battery Park (NR, 2023 for PG)	NCC	17.9	PG, Trails/Paths	1 Delaware St, New Castle, DE 19720
16	Rogers Manor Park (NR)	NCC	8.5	Baseball/Softball Field, Basketball Court, Multipurpose Fields, PG, Soccer Field, Tennis Court	441 Moores Lane, New Castle DE, 19720
17	Townsend Municipal Park (2006)	NCC	11.5	Basketball Court, Fitness Equipment, Pickleball Court, PG, Skatepark, Trails/Paths	0 Edgar Rd, Townsend, DE 19734
18	Eden Park (1890)	NCC	13.43	Basketball Courts, Football Fields, Multipurpose Fields, PG, Swimming Pools	900 New Castle Ave, Wilmington, DE 19801
19	Brown Burton Winchester Park (1917)	NCC	55.06	Baseball Fields, Basketball Courts, Multipurpose Fields, PG, Swimming Pools	2313 N Locust Street, Wilmington, DE 19802
20	Talley Day Park (1970)	NCC	37	Baseball/Softball Field, Basketball Court, Delaware Greenways, Multipurpose Fields, PG, Soccer Field, Tennis Court, Trails/Paths	1308 Foulk Rd, Wilmington DE 19803
21	Powell Ford Park (NR)	NCC	200	Baseball/Softball Field, PG	1000 Kiamensi Rd, Wilmington, DE 19804
22	Kosciuszko Park (1886)	NCC	7.08	Basketball Courts, PG, Tennis Courts	1320 Beech Street, Wilmington, DE 19805
23	Father Tucker Memorial Park (1910)	NCC	3.68	Baseball Fields, PG	1800 W 10th Street, Wilmington, DE 19805
24	Cool Springs Park (1862)	NCC	14.7	Fitness Equipment, Multipurpose Space, PG, Trails/Paths	1001 N Van Buren St, Wilmington, DE 19806
25	Delcastle Recreational Park (1974)	NCC	400	Baseball/Softball Field, Basketball Court, Fitness Equipment, Football Field, Multipurpose Fields, PG, Soccer Field, Street Hockey, Tennis Court, Trails/Paths	2920 Duncan Rd, Wilmington, DE 19808
26	NCC Delpark Manor Park (NR)	NCC	7	Baseball/Softball Field, Basketball Court, PG, Trails/Paths	2010 St James Church Rd, Wilmington, DE 19808
27	River Road Park (NR)	NCC	8.5	Baseball/Softball Field, Multipurpose Fields, PG, Soccer Field	610 River Rd, Wilmington, DE 19809
28	Bonsall Park (NR)	NCC	17.8	Baseball/Softball Field, Basketball Court, PG, Soccer Field, Tennis Courts, Trails/Paths	3000 Silverside Rd, Wilmington, DE 19810
29	Tidbury Creek County Park (NR)	KC	30	Multipurpose Fields, PG, Softball Fields, Trails/Paths	2233 S State St, Dover, DE 19901
30	Dover Park (1974)	KC	28.2	Basketball Courts, Disc Golf Courses, PG, Pickleball Courts, Shuffleboard Courts, Softball Field, Tennis Courts, Trails/Paths	1210 White Oak Rd, Dover, DE 19901
31	Silver Lake Park (1917)	KC	182	Fitness Equipment, PG, Trails/Paths	300 Washington St Dover, DE 19901
32	Mallard Pond Park (2014)	KC	6.07	PG, Trails/Paths	45 Marsh Creek Ln, Dover, DE 19904
33	Brecknock County Park (1996)	KC	86	Baseball/Softball Fields, Fitness Equipment, Football Field, Horseshoes Pits, Multipurpose Fields, PG, Sand Volleyball Courts, Sports Pavilion, Trails/Paths, Youth Activity Center	80 Old Camden Rd, Camden, DE 19934
34	Browns Branch County Park (2006)	KC	78	Baseball/Softball Fields, Fitness Equipment, Horseshoes Pits, Multipurpose Fields, PG, Sand Volleyball Courts, Soccer Field, Trails/Paths	1415 Killens Pond Rd, Harrington, DE 19952
35	Tony Silicato Memorial Park & Can-Do PG (2006)	KC	31	Disk Golf Course, Multipurpose Fields, PG, Soccer Fields, Trails/Paths	101 Delaware Veterans Blvd, Milford, DE 19963
36	Big Oak County Park (2003)	KC	90	Baseball/Softball Fields, Fitness Equipment, Multipurpose Fields, PG, Rock Climbing Wall, Sports Pavilion, Trails/Paths, Youth Activity Center	417 Big Oak Rd, Smyrna, DE 19977
37	Lake Como Recreation Area (1956)	KC	35	PG, Swimming Beach Area	420 S Dupont Blvd, Smyrna, DE 19977
38	Schutte Park (2001)	KC	71	Baseball/Softball Fields, John W. Pitts Recreation Center, Multipurpose Fields, PG, Trails/Paths	10 Electric Ave, Dover, DE, 19904
39	Frankford Community Park (2012)	SC	33	Basketball Court, Fitness Equipment, Multipurpose Field, PG, Soccer Practice Goals, Trails/Paths, Volleyball Court,	30 Clayton Ave, Frankford, DE 19945
40	John West Park (NR)	SC	4.5	Fitness Equipment, PG, Trails/Paths	32 West Ave, Ocean View, DE 19970
41	Roger C. Fisher Laurel River Park (2001)	SC	2.8	Canoeing, Fishing, Kayaking, Motorized Boating, Exercise Circuit, PG	W 6th St, Laurel, DE 19956
42	Lewes CanalFront Park (2009)	SC	14.6	Baseball/Softball Fields, Canoeing, Kayaking, Fishing, Multipurpose Field, Pickleball Court, PG, Softball Field, Tennis Court	211 Front St, Lewes, DE 19958
43	George H.P. Smith Park (NR)	SC	15.1	Fishing, Horseshoe Pit, Multipurpose Fields, PG, Trails/Paths	Dupont Ave, Lewes, DE 19958
44	Cupola Park (1976)	SC	10	Canoeing, Kayaking,	end of Morris St, Millsboro, DE 19966
45	Milton Memorial Park (1939)	SC	4	Canoeing, Kayaking, Fishing, PG, Trails/Paths	113 Union St, Milton, DE 19968
46	The Grove Park (1963)	SC	2.4	Canoeing, Kayaking, Fitness Circuit, PG, Shuffleboard Courts, Trails/Paths	3 Grove St, Rehoboth Beach, DE 19971
47	Soroptimist Park/ Walking Trail (2001)	SC	2.8	PG, Trails/Paths	1100 Middleford Rd, Seaford, DE 19973
48	Marvel Square Park (NR)	SC	NR	Grassy area, open space, pavilion (optional), PG	207 Franklin St, Milford, DE 19963
49	Jay’s Nest & Sports Complex (2002)	SC	26	Baseball/Softball Fields, Football Fields, Horseshoe Pits, Multipurpose Field, PG, Soccer Fields, Trails/Paths	490 N Market St Ext, Seaford, DE 19973
50	Swann Keys Park	SC		PG; Mini-golf; Basketball; Multi-purpose courts (Pickleball/Tennis); Volleyball; Pool & kids’ splash zone; Shuffleboard; Picnic pavilion; Horseshoe pits	37689 Swann Dr, Selbyville, DE 19975

Note. NR = not reported on park website or cannot be determined; NCC = New Castle County; KC = Kent County; SC = Sussex County; PG = playground. † Delaware’s first PG designed for children with autism spectrum disorder.
